# The Fundamental Nature of Motives

**DOI:** 10.3389/fnins.2022.806160

**Published:** 2022-01-28

**Authors:** Arto Annila

**Affiliations:** Department of Physics, University of Helsinki, Helsinki, Finland

**Keywords:** free energy, force, quantum, photon, statistical physics, thermodynamics, utility

## Abstract

Decision-making is described as a natural process, one among others, consuming free energy in the least time. The thermodynamic tenet explains why data associated with decisions display the same patterns as any other data: skewed distributions, sigmoidal cumulative curves, oscillations, and even chaos. Moreover, it is shown that decision-making is intrinsically an intractable process because everything depends on everything else. However, no decision is arbitrary but bounded by free energy, such as resources and propellants, and restricted by mechanisms like molecular, neural, and social networks. The least-time maximation of entropy, equivalent to the minimization of free energy, parallels the optimization of subjective expected utility. As the system attains a state of balance, all driving forces vanish. Then there is no need or use to make further decisions. In general, the thermodynamic theory regards those decisions well-motivated that take into account forces, i.e., causes comprehensively in projecting motions, i.e., consequences.

## Introduction

Making sense of data takes a theory. However, the data itself is already theory-laden because theories influence data acquisition (Kuhn, 1962). Thus, the mere attempt to make sense of decision-making also narrows experiments and biases interpretations toward regarding behavior as rational—eventually focusing on what rationality means.

A theory (Greek *theôría* “a looking at”) provides a perspective. The view can be held true as long as its explanations are in line with observations. Conversely, the theory is irreparably faulty if empirical evidence contradicts the founding axiom. Then another tenet is needed. In contrast to the axiomatic theory, an effective theory, i.e., a model of data, cannot be proven wrong with the data it models.

Although these prerequisites and qualities of a theory (Popper, 2002) are acknowledged, it is not apparent on what axioms contemporary research of decision-making rests. On the one hand, studies of mutated mice, fruit flies, and nematodes correlate neural networks and molecular mechanisms with behavior to dazzling detail (Yapici et al., 2014; Tanimoto and Kimura, 2019). Yet, the central aspect of science, causality, remains unclear: Do the molecular structures and neural networks facilitate or force behavior? What fundamentally motivates decisions? Why do we decide the way we do?

On the other hand, thermodynamics, the grand theory of nature, provides an unprecedented perspective to motives and motions: forces are causes and changes in motions are consequences. Yet, textbook thermodynamics does not say what in substance embodies motives, intents, drives, etc., as well as motions, actions, emotions, etc. Consequently, the theory cannot be put into practice (Greek *praxis* “action, doing”). As long as fundamental properties, notably, energy and time, are not explicitly and exactly associated with firm physical entities, theorizing cognition (Wolpert, 2004; Friston, 2010; Ortega and Braun, 2013; Annila, 2016; Roy, 2016; Deli et al., 2018, 2021) is modeling, for example, in terms of game-theory (Jaynes et al., 1985; Anttila and Annila, 2011; Babajanyan et al., 2020) or modern physics (Busemeyer and Bruza, 2012; Roy, 2015; Rastmanesh and Pitkänen, 2021). Thus, a concrete axiom is needed to conceptualize natural processes, such as decision-making.

In the quest for a universal theory, Ludwig Boltzmann strived to derive thermodynamics from the atomistic axiom but failed (Boltzmann, 1905). As Boltzmann’s contemporaries already remarked (Loschmidt, 1876; Zermelo, 1896), the renowned many-body theory applies only to a stationary state, where motions are recurrent rather than irreversible. Thus, expressing thermodynamics in evolutionary terms had to wait until our time (Annila and Salthe, 2010).

So, the issue was not Boltzmann knowing the elemental constituent explicitly, for his atomistic theory is scale-free. Instead, the issue was him erroneously approximating motions of atoms as random rather than forced. Curiously, Boltzmann had adopted the random-walk, i.e., Gaussian approximation, leading to the Boltzmann distribution, from Adolphe Quetelet, a pioneer of social physics (Ball, 2014). Hence, modeling society in statistical terms led to modeling molecular ensemble rather than making sense of both in thermodynamic terms.

Admittedly, the stochastic approximation reproduces data at thermodynamic balance, where forces vanish and nothing happens. However, natural processes, including information processing, are all about happening, i.e., flows of quanta (Annila, 2021). Imbalance is a cause; changes in motion are consequences. Thus, data distribute about the mean in a characteristically skew manner rather than symmetrically (Contreras-Reyes, 2021). Distributions cumulate along sigmoid curves that follow mostly straight lines, i.e., power laws on log-log plots (Limpert et al., 2001; Newman, 2005; Bennet and Bennet, 2008; West, 2017). Thus, empirical evidence against random processes is undeniable. Hence, textbook thermodynamics (Callen, 1991) is not a proper starting point for sense-making, whereas non-equilibrium thermodynamics seems perfect (Mäkelä and Annila, 2010).

Weighed by facts and figures, decision-making neither exhibits nor entails anything special. From molecules to man and cells to society, data related to decision-making, when plotted without legends and labels, are similar to any other process (Wohrer et al., 2013; Altman, 2015). Thus, the ubiquity of patterns speaks for a universal law of nature making sense of decision-making too.

In the following, the thermodynamic theory of decision-making is derived from the atomistic axiom. Results are contrasted with conventional accounts on decision-making. Finally, inferences drawn from the thermodynamic theory about decision-making are discussed.

## From Axiom to Theory

Since it is unclear what all decision-making encompasses, a theory is best founded on an all-inclusive axiom. Parmenides, Galileo, Newton, and Boltzmann argued that everything must ultimately comprise the same elemental constituent, for otherwise, a change of any kind would be impossible (Pullman and Reisinger, 2001). While it is not necessary to know the fundamental element explicitly to formulate the theory, it is still enlightening to reason, as Gilbert Lewis did, that everything ultimately comprises quanta of light (Lewis, 1926). The conjecture makes perfect sense, as, for instance, chemical reactions, underlying both natural and artificial information processing, dissipate photons in the form of heat.

Assuming that everything consists of the same basic element, it is possible to express the state of any system, may that system be a cellular metabolic network, neural network, or social network involved in decision making. Once the equation of state is written, the equation of change is obtained by differentiation with respect to time (Sattin, 2018). The equation of change reveals the fundamental nature of motives. Also, the subjective and resource-limited as well as irreversible and non-determinate characteristics of decision-making become clear.

### The Equation of State

**FIGURE 1:**
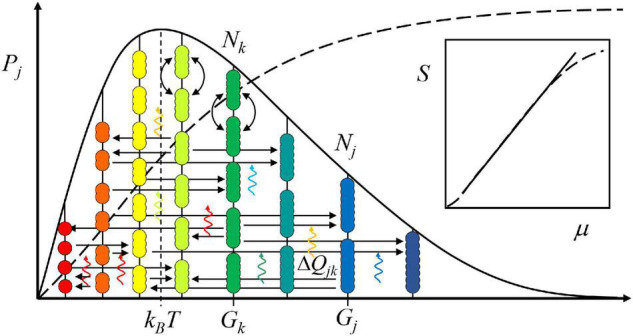
Assuming everything comprises the same fundamental elements, the quanta, a decision-making system, as any system, can be pictured in terms of an energy level diagram. The entities in numbers *N*_*k*_, with the same energy *G*_*k*_, are on the same level. The bow arrows portray their mutual exchange, which causes no change in the average energy of the system, *k_*B*_T*. The horizontal arrows correspond to transformations moving the entities from one level to another. For example, in a chemical reaction, starting materials, *N*_*k*_, transform into products, *N*_*j*_. The vertical wave arrows denote the quanta of light that couple to the transformations by entering the system from the environment or vice versa. Since the quanta carry energy, Δ*Q*_*jk*_, all events, as flows of quanta, move the system and its surroundings toward thermodynamic balance. When the surroundings are higher in energy than the system, the system evolves toward higher average energy and the surrounding systems toward lower average energy, and vice versa. The cumulative probability distribution curve (dotted line) is a sigmoid. When its logarithm, entropy, *S*, is plotted as a function of (chemical) potential energy, μ, it mainly follows a power law, i.e., a straight line on the logarithm-logarithm scale (inset).

The atomistic axiom allows describing the state of any system by a general energy level diagram (Figure 1). The diagram, in turn, can be mathematized into the equation of state using the standard procedure of statistical mechanics (Gibbs, 1902; Sharma and Annila, 2007): First, the probability of a given entity’s existence is inferred. Second, the probability of a population holding the given entities is deduced. Finally, the probability of a system housing all populations is reasoned. The view is systemic, hence subjective.

It is worth emphasizing that in statistical mechanics, the probability enumerates ingredients of existence, not arbitrates chances. For example, options and risks are gauged by what it takes for them to happen, not assigned with likelihoods.

First, consider an entity, for example, a neurotransmitter molecule, by asking what it takes for that entity, labeled with *j*, of energy *G*_*j*_ to exist. Clearly, substrates, indexed with *k*, in numbers *N*_*k*_ each with energy *G*_*k*_ are necessary. If any one of the vital ingredients were missing altogether, the entity *j* could not exist. Thus the probability of existence _1_*P*_*j*_ is a product ∏*_*k*_* over the substrates. Moreover, the probability depends on the energy difference -Δ*G*_*jk*_ between the *j*- and *k*-entities relative to the average energy of the system *k_*B*_T*. Besides material ingredients, the photons of energy Δ*Q*_*jk*_ matching the difference are needed for the *jk*-transformation to happen. For example, chemical syntheses are either endo- or exoergic transformations. Adopting Boltzmann’s and Gibbs’ exponential form of energy, valid for a statistical system, the probability of the *j*-entity is


(1) Pj1=∏k=1Nk⁢exp⁢[(-△⁢Gjk+i⁢△⁢Qjk)/kB⁢T]

where the prefix *i* explicitly distinguishes radiation from matter, in physics terms vector potential from scalar one.

Second, consider the probability *P*_*j*_ of a population of *j*-entities, for example, a neurotransmitter population. If any *j*-entity in the population of *N*_*j*_ was missing altogether, *P*_*j*_ = 0. Thus,


(2) Pj=∏j=1Pj1/Nj!=PjNj1/Nj!,

where the division by the factorial *N*_*j*_! is in place because the order among indistinguishable entities makes no difference.

Finally, consider the total probability *P* of a system housing all *j*-populations, for example, neurons and neurotransmitters. Again, if any *j*-population was missing altogether, *P* = 0. Thus,


(3) $$\text{P}=\prod_{j=1}{\text{P}}_{j.}$$

Customarily, entropy, as the logarithm of probability multiplied with Boltzmann’s constant,


(4) $$\text{S}={\text{k}}_{B}\,\sum_{j=1}{\text{lnP}}_{j}\approx \frac{1}{\text{T}}\,\sum_{j,k=1}{\text{N}}_{j}\,\left(-△\,\mu_{\mathrm{jk}}+\text{i}\,△\,{\text{Q}}_{\mathrm{jk}}+{\text{k}}_{B}\,\text{T}\right)$$

is used as the measure of state (Figure 1, inset) because *S* is additive, while *P* is a product. The shorthand notation *μ_*j*_* = *k_*B*_T*ln[*N*_*k*_(exp(*G*_*j*_/*k_*B*_T*)] stands for (chemical) potential. The approximation following from ln*N*_*j*_! ≈ *N*_*j*_ln*N*_*j*_ – *N*_*j*_ is suitable for a statistical system.

The equation of state shows imbalance in the form of free energy −Δ*μ_*jk*_* + *i*Δ*Q*_*jk*_ ≠ 0. The system must move. Conversely, at balance, entropy reduces to *S* = *k*_*B*_∑*N*_*j*_. This familiar form from Boltzmann’s statistical mechanics essentially enumerates all entities in the steady-state system, e.g., molecules in a chemical reaction mixture at balance and neurons in a network in a dynamic balance. So, consistently with common sense, causes, i.e., forces, free energy terms, demand decisions. And contrariwise, as the saying goes, “When it is not necessary to make a decision, it is necessary not to make a decision.”

Undoubtedly, any given state of a decision-making system involves numerous factors. Still, the atomistic equation (Eq. 4) includes all of them. Thus, whatever minutia, tipping a decision one way or another, is taken into account formally, while in practice, there may not be means to pinpoint it. So, the objective is not to quantitatively predict what will be decided but to make qualitatively clear why the decision will be made. Namely, a force, however fleeting, motivates decisions of any kind.

### The Equation of Change

The equation of change, expressly a decision, is obtained as a time derivate of *S* multiplied with *T*


(5) $$\text{T}\,\frac{\text{dS}}{\text{dt}}=\text{T}\,\sum_{j=1}\frac{\text{dS}}{{\text{dN}}_{j}}\,\frac{{\text{dN}}_{j}}{\text{dt}}=\sum_{j,k=1}\frac{{\text{dN}}_{j}}{\text{dt}}\,\left(-△\,\mu_{\mathrm{jk}}+\text{i}\,△\,{\text{Q}}_{\mathrm{jk}}\right)$$

using the chain rule. The least-time free energy consumption, operating across all levels, bonds subsystems into the system, for example, molecules to a neuron and neurons to a network. Conversely, failure in interacting, say, cohering, splits the system apart. The total energy keeps changing until the thermodynamic balance is attained. At the stationary state, there is no power *TdS*/*dt* = 0. Thus, according to the scale-free theory, decision-making at any level is fundamentally motivated by the quest for balance with the surrounding systems so that all forms of free energy −Δ*μ_*jk*_* + *i*Δ*Q*_*jk*_ have been consumed.

At first sight, the fundamental motive of making decisions to gain balance may seem counterintuitive. Is not decision-making about making progress, not ending up with stagnation? Indeed, but progress calls for motive forces. A non-optimal state is a state of imbalance.

As the grand sum over entities (Eq. 5) defines the system through its interactions, the scope of decision-making becomes apparent. A decision is ranked by the associated change in free energy. Explicitly, a good decision takes into account free energy widely, i.e., broadmindedly, while a bad one narrowly. The good decision opens up substantial consumption of free energy, whereas the bad one closes opportunities.

According to the conservation of the elemental constituents, the rate of change in a population


(6) $$\frac{{\text{dN}}_{j}}{\text{dt}}=\frac{1}{{\text{k}}_{B}\,\text{T}}\,\sum_{k=1}\sigma_{\mathrm{jk}}\,\left(-△\,\mu_{\mathrm{jk}}+\text{i}\,△\,{\text{Q}}_{\mathrm{jk}}\right)$$

is proportional to free energy by mechanisms of energy transduction σ_*jk*_ (Kondepudi and Prigogine, 1999). Such decision-making mechanisms are, for instance, neural networks, which are systems themselves resulting from natural processes. Also, procedures and protocols facilitate rather than force decision-making.

Accordingly, natural processes *naturally select* effective mechanisms to consume free energy in the least time. However, the bias for efficiency through established but inapt mechanisms directs the system to a non-optimal course because relevant forces are hardly sensed without appropriate means. For example, decision-making proceeds rapidly through developed neural networks and established social connections to a conventional outcome even when the decision should challenge convention or authority.

The rate equation reveals the non-determinate character of natural processes. Since *dN*_*j*_/*dt* depends on free energy that, in turn, depends on *N*_*j*_, the variables cannot be separated. Thus, the equation of change (Eq. 5) cannot be solved. The motion remains fundamentally intractable. However, the future is not all arbitrary as courses are bounded by free energy. Thus, the consequences of decision-making are truly unpredictable only as much as they bring forth unforeseeable forces. Again, the key to good decision-making is sensitivity to diverse forces. So, while the processes given by Eqs. 5 and 6 cannot be solved exactly, they can be simulated approximately (Iacus, 2009).

It is worth emphasizing the perspective on the forces and motions through Eqs. 1–6 is systemic, i.e., subjective. For example, had a neurotransmitter concentration been larger, the neuron might have fired with consequences. Likewise, had a decision-maker been more sensitive to various forces, the decision might have differed from the one made. Especially, the greatest forces embedded in values, morals, and trust might be momentarily neglected but turn out to be compelling in the long run (Harris, 1980; Gold et al., 2011).

### The Ubiquitous Patterns

When the change in energy is small compared with the average energy —(−Δ*μ_*jk*_* + *i*Δ*Q*_*jk*_)/*k_*B*_T* — < < 1, the variation *n* in *j* is small, i.e., *n* < < *j*, around a representative, an average factor *ϕ_*j*_* = *N*_*j*_exp(*G*_*j*_/*k_*B*_T*). Then the factors, given in logarithmic terms, distribute as


(7) $$\text{ln}\,\phi_{j-n\,\cdots \,j+n}=\text{ln}\,\phi_{j}+\sum_{n}\text{nln}\,\phi_{1}$$

where ln*ϕ_*j*_* = *j*lnϕ_1_ expressed in terms of the basic factor *f*_1_. Thus, the approximately lognormal distribution (Gaddum, 1945; Aitchison and Brown, 1963; Crow and Kunio, 1988) extends further out than the normal distribution. This is to say, rare outcomes are not as unlikely as rated by standard deviations (Taleb, 2008). For instance, exceptionally good or bad decisions are not that uncommon.

The sigmoid shape of the cumulative curve can be inferred from the rate equation (Eq. 6). Initially, when forces are big, it can be assumed that mechanisms limit the free energy consumption


$$\frac{\text{d}}{\text{dt}}\,\frac{1}{{\text{k}}_{B}\,\text{T}}\,\sum_{k=1}\left(-△\,\mu_{\mathrm{jk}}+\text{i}\,△\,{\text{Q}}_{\mathrm{jk}}\right)$$

(8) $$=\frac{{\text{dN}}_{j}}{\text{dt}}\,\frac{\text{d}}{{\text{dN}}_{j}}\,\frac{1}{{\text{k}}_{B}\,\text{T}}\,\sum_{k=1}\left(-△\,\mu_{\mathrm{jk}}+\text{i}\,△\,{\text{Q}}_{\mathrm{jk}}\right)\approx \sum_{k=1}\sigma_{\mathrm{jk}},$$

which reduces to


(9) $$\frac{{\text{dN}}_{j}}{\text{dt}}=\sum_{k=1}\sigma_{\mathrm{jk}}\,{\text{N}}_{j}$$

using *dμ_*j*_*/*dN*_*j*_ = *d*(*G_*j*_* + *k_*B*_T* ln*N*_*j*_)/*dN*_*j*_ = *k_*B*_T*/*N*_*j*_, as *μ_*k*_*, *Q_*j*_*, and *Q*_*k*_ do not explicit depend on *N*_*j*_. Thus, the integrated Eq. 9 shows that the initial rate of change is exponential. Conversely, when free energy is about to vanish, the sigmoidal curve flattens out almost exponentially.

At the intermediate region, the cumulative curve follows a power law, as is seen by expressing *N*_*j*_ as a product of its constituents *N*_*k*_ that, in turn, all can ultimately be expressed as products of the elemental constituents *N*_1_. Thus, the change


(10) $$\frac{{\text{dN}}_{j}}{\text{dt}}=\text{j}\,\alpha_{j}\,{\text{N}}_{1}^{j-1}\,\frac{{\text{dN}}_{1}}{\text{dt}}=\text{j}\,\frac{{\text{N}}_{j}}{{\text{N}}_{1}}\,\frac{{\text{dN}}_{1}}{\text{dt}}⟹\frac{{\text{dN}}_{j}}{{\text{N}}_{j}}=\text{j}\,\frac{{\text{dN}}_{1}}{{\text{N}}_{1}}$$

when integrated, follows a power law. The free energy factor


(11) $$\alpha_{j}=\prod_{\mathrm{mn}}\text{exp}\left[\left(-△\mu_{\mathrm{mn}}+i△{\text{Q}}_{\mathrm{mn}}\right)/{\text{k}}_{B}\text{T}\right],$$

indexing all transformations 1 ≤ *m, n* ≤ *j* extending from the elemental constituents *N*_1_ to the product *N*_*j*_, is approximately constant. In other words, the energy level diagram keeps its form throughout the intermediate region.

When the approximation —(−Δ*μ_*jk*_* + *i*Δ*Q*_*jk*_)/*k_*B*_T* — << 1 holds, the system consumes free energy in a trend-like manner, often approximated by a power law or sigmoid curve (Strogatz, 2018). It means, for example, that mature neural circuitry does not sway easily and highly integrated society does not end up in turmoil at all of a sudden. Decisions are quite predictable.

Conversely, when the approximation fails, changes are abrupt. This is typical of a small system. For example, early stage decisions set the future course almost irrevocably. Thus, a nascent neuronal network is prone to biases. Also, a large system may experience a sudden change when the supply of free energy changes substantially. The supplies may run out or mechanisms to tap into them may break down extensively. Such an incidence could be a shock, a trauma, or a metabolic failure.

### The Scope of Thermodynamic Theory

Thermodynamics resulting from the atomistic axiom may seem scant in concepts to cover the richness of decision-making. However, the high-level concepts relevant to a given case can be constructed from the elemental ones by the scale-free theory. For example, atoms make molecules in photon-coupled transformations, molecules cells, cells tissues, tissues organisms, and so on. Alternatively, high-level abstractions can be decomposed into the basic concepts of atomism. And if not, then the abstraction is deemed to be without unambiguous correspondence with reality.

While information processing at the molecular level, essentially chemical reactions, is clearly within the scope of thermodynamics, processing at the neural level can also be described likewise. Neurons make contacts as atoms make bonds. Thus, a neural network, just as a molecule, displays emergent properties. Also, a social network, just as a neural network, materializes with novel functions by linking nodes. This is to say, the thermodynamic theory of open, evolving systems is not reductionism. Instead of permuting existing entities, ingredients from the surroundings, most notably photons, integrating into transformations are taken into account (Tuisku et al., 2009).

#### Thermodynamic Perspective

Understanding decision-making as one among natural processes is perhaps best absorbed by comparing it with conventional accounts on decision making. On the one hand, normative, rational decision theory defines maxims of how decision-makers should decide; on the other, descriptive, psychological decision theory deduces rules from the spectrum of how individuals actually decide. In a sense, thermodynamics provides a perspective on the proficiencies and deficiencies of the conventional tenets.

### Normative Theory

Utility is the central concept in rationalizing behavior in general and decision-making in particular. However, its essence is vague. From the thermodynamic viewpoint, utility is subsumed into free energy. Without any utility, i.e., driving force, there is no need to make any decisions. Also, in case only one force is in command, the subject obeys without choice. Thus, deciding is conceptualized as choosing among alternatives the option that maximizes utility. In thermodynamic terms, the optimal choice is the one maximizing entropy, equivalently minimizing free energy in the least time (Eq. 5).

The least-time temporal aspect implies optimization of the whole process. However, as decisions depend on the consequences of past decisions, the natural process is fundamentally intractable. Mathematically speaking, the process is non-integrable because the limit of integration moves as integration proceeds. For example, initial, tentative decisions outline the bulk of decisions homing in on a goal, and final decisions perfect the achievement. Thus, the series results in the characteristic, sigmoidal curve (Eqs. 9 and 10). The sequence of events, where quanta carry energy on their periods of time, produces a passage of time (Greek *chronos*). In turn, timing (Greek *kairos*) corresponds to a critical event, a turning point that directs subsequent flows of quanta along particular paths.

According to the normative decision theory, a rational agent is deemed to maximize the subjective expected utility. In fact, an objective utility would be a misnomer, an illusion because free energy is invariably associated with the state of a subject, such as the state of awareness. As chemical reactions depend on conditions, i.e., on states, awareness ultimately emerges from molecular, neural, physiological, etc., states. Also, biases, such as cognitive ones, correspond to various states, fundamentally enumerated by systemic probabilities (Eq. 3).

From the thermodynamic perspective, the rational decision theory sums major forces into the utility function and ascribes circumstantial factors to biases. For example, prejudice, confirmation bias, repetition bias, and cognitive inertia relate to mechanisms (Eq. 6) that have perfected processing supporting information while failing when facing opposing information. Likewise, a metabolic system has adapted to a diet from which deviations present problems. Also, an economy has advanced to process specific resources whereas struggling when diverting to other sources.

Furthermore, framing a decision problem (Tversky and Kahneman, 1981) relates to choosing the forces at play. For example, a social setting, let alone whole culture and natural circumstances, impose tremendous forces and engage monstrous machinery biasing decisions. In this manner, the cognitive theory (Piaget, 1976) also regards decision-making as a continuous process in interaction with surroundings. Akin to thermodynamics, metabolic theory aims at keeping a running tally of flows matter and radiation through the whole biosphere to account for the observed scale-free patterns (Schramski et al., 2015).

Despite its conceptually appealing character, the predictive power of normative theory is challenged in practice. For one thing, a single utility function cannot model the free energy function housing every quantum (Eq. 5). Exponential and power-law models are good but not perfect approximations of the free energy consumption (Eqs. 9 and 10). Deviations from theoretical expectations are pronounced when rare events of long-tailed distributions manifest themselves. Moreover, the expected utility may present acausal terms, whereas the free energy terms always correspond to causes. Furthermore, when free energy is comparable to bound energy, bifurcations, oscillations, and even chaotic behavior follow. Thus, decisions can be highly unpredictable when everything is at stake.

The normative decision theory does not acknowledge that decision-making as a natural process is intrinsically non-deterministic (Eq. 6). On the contrary, it aims at predicting decisions. However, as the system makes decisions in response to forces imposed by its surroundings, the decision causes changes in the surroundings, and so on. Thus, the free energy consumption cannot be known beforehand exactly, only anticipated. Instead of explicitly taking into account the non-determinate and subjective characteristics of decision-making, decisions are said to be based on expectations and beliefs. But, of course, the expectations and beliefs themselves emerge from past incidences, say, decisions. In contrast, thermodynamics includes in the state equation (Eq. 4) the whole history embodied in the mature structures, e.g., ranging from full-fledged cognitive faculty to developed institutions of society.

While the quest for maximizing utility is ascribed to rational agents, the least-time consumption of free energy is a universal imperative, irrespective of agency and its rationality, from chemical reactions to societal transactions. A neuron integrates inputs to output by the same principle as a society integrates opinions to actions. Rationality too is thus rated by the least-time free energy consumption.

### Descriptive Theory

Paradoxes of decision-making, where a given utility function fails to predict behavior, have led to an antidote to normative, rational theory, coined as descriptive, psychological theory. Customarily, deviations from expectations are attributed to differences between the real and rational agents. However, rationality, and irrationality for that matter, are just as elusive concepts as utility itself (Ariely, 2008; Kahneman, 2011).

The thermodynamic perspective on decision-making clarifies, for instance, that preference for certainty and aversion of losses (Kahneman and Tversky, 1979) show that free energy is a non-linear, fundamentally a discontinuous function of the quantized process. Moreover, laboratory tests can be prepared to go against one’s life experience, i.e., holistic reasoning about causes and consequences. Tests can also be tailored to deviate from the characteristically non-determinate courses of natural processes anticipated by participants. To trick is to distract.

Shortage of information, just as surplus, may lead to astray judged by a second thought. In thermodynamic terms, the system had not enough resources to consider all forces before deciding. Alternatively, the system did, in fact, consider more but in an unconscious manner. For example, decisions are made by gut feeling when there is not enough time and energy to weigh factors, pros and cons, and evaluate scenarios explicitly. In a crisis, immediate action offsets investing in decision-making. When in plight, effortless, and straightforward ways are preferred over effortful and sophisticated ways (Fiske and Taylor, 1991). In any case, free energy is limited, i.e., rationality is bounded (Simon, 1955).

Also, paralysis by analysis can be rationalized in thermodynamic terms. Neverending analysis corresponds to a metastable state. The system is balancing between options. Gains in free energy consumption by one decision or another are marginal or seemingly incommensurate, hence providing no unambiguous impetus for one or the other. Since natural processes are intractable, it is impossible to know beforehand under which circumstances paralysis occurs.

In the end, the descriptive decision theory, like the rational one, lacks universality. A model accounts for a phenomenon but fails to generalize for phenomena. From the thermodynamic perspective, a natural process’ subjective and non-determinate characters prevent deriving a universal model. Even so, decision-making is not arbitrary but produces universal patterns since it is bounded by free energy, channeled by mechanisms, and directed by the least-time imperative.

## Discussion

Decision-making implies the existence of free will (O’connor and Franklin, 2021). However, the concept seems elusive. By the thermodynamic theory, free will corresponds to free energy (Annila, 2016). For the first, decisions are relevant as much as they can be realized. Those in power decide on how free energy is consumed, i.e., what will happen. For the second, decision-making itself consumes resources, e.g., by acquiring and processing information. Hence decisions are invariably bounded by free energy, say, free will. Thus, determinism contradicts thermodynamics that complies with common sense verbalizing it in exact and comprehensive terms.

Moreover, the thermodynamic theory clarifies that decision-making, as a natural process, channels through mechanisms. Therefore molecular, neural, organismal, societal, environmental, etc., structures influence decision-making. In other words, it is hard to think outside the box as it entails emerging with new structures and abandoning old ones. Customarily, this mode of consuming free energy is referred to as creativity, at times dissidence.

Despite its comprehensiveness, the thermodynamic theory may not meet the expectations often associated with a theory, say, predicting a decision. However, the theory clarifies that natural processes are intrinsically intractable because everything depends on everything else. As variables cannot be separated to solve the equation of motion, the future lies beyond precise predictions. Conversely, the theory clarifies that the future opens up from consuming resources through structures inherited from the past. From this perspective, the whole cognitive faculty, decision-making at its core, is geared up projecting ourselves from the past into the future. Thus, consciousness adheres to the present (Fingelkurts and Fingelkurts, 2014), where fluxes of quanta route through one or another path (Tuisku et al., 2009; Annila, 2021).

The thermodynamic theory rates decisions by their least-time free energy consumption quantitatively and unambiguously. The holistic criterium maintains that the more forces are taken into account, the better the decision. At the neural level, this manifests itself in the evolution toward larger neural networks. At the societal level, it means progression from exclusive uniformity to cohesive diversity. Conversely, the inability to put oneself in someone else position, or groupthink, runs the risk of making poor decisions. To go against the greatest forces, presenting themselves as values, morals, and trust, is particularly devastating as they hold the system together. Disrupting global unity by objecting to natural forces results in catastrophic consequences, e.g., climate change and loss of biodiversity.

In any case, rating a decision comes with the benefit of hindsight. The course of events exposes overlooked forces. However, as all processes follow the same principle, history offers us lessons. Thus, the failure to foresee consequences follows from not seeing causes, i.e., forces by relating the present case with past incidences and not drawing parallels across the whole hierarchy of existence.

In short, thermodynamics is not thermodynamic decision theory but the theory subsuming decision-making as a natural process. The obtained comprehension puts us as decision-makers in an unprecedented perspective of responsibility—everything depends on everything else, irresistibly and irreversibly.

## Data Availability Statement

The original contributions presented in the study are included in the article/supplementary material, further inquiries can be directed to the corresponding author/s.

## Author Contributions

AA contributed to the conception, elaboration, and completion of the study from the first draft of the final manuscript.

## Conflict of Interest

The author declares that the research was conducted in the absence of any commercial or financial relationships that could be construed as a potential conflict of interest.

## Publisher’s Note

All claims expressed in this article are solely those of the authors and do not necessarily represent those of their affiliated organizations, or those of the publisher, the editors and the reviewers. Any product that may be evaluated in this article, or claim that may be made by its manufacturer, is not guaranteed or endorsed by the publisher.
